# Targeting the Endocannabinoid System for Prevention or Treatment of Chemotherapy-Induced Neuropathic Pain: Studies in Animal Models

**DOI:** 10.1155/2018/5234943

**Published:** 2018-07-25

**Authors:** Willias Masocha

**Affiliations:** Department of Pharmacology and Therapeutics, Faculty of Pharmacy, Kuwait University, Safat 13110, Kuwait

## Abstract

There is a scarcity of drugs to either prevent or properly manage chemotherapy-induced neuropathic pain (CINP). Cannabis or cannabinoids have been reported to improve pain measures in patients with neuropathic pain. For this review, a search was done in PubMed for papers that examined the expression of and/or evaluated the use of cannabinoids or drugs that prevent or treat established CINP in a CB receptor-dependent manner in animal models. Twenty-eight articles that fulfilled the inclusion and exclusion criteria established were analysed. Studies suggest there is a specific deficiency of endocannabinoids in the periphery during CINP. Inhibitors of FAAH and MGL, enzymes that degrade the endocannabinoids, CB receptor agonists, desipramine, and coadministered indomethacin plus minocycline were found to either prevent the development and/or attenuate established CINP in a CB receptor-dependent manner. The studies analysed suggest that targeting the endocannabinoid system for prevention and treatment of CINP is a plausible therapeutic option. Almost 90% of the studies on animal models of CINP analysed utilised male rodents. Taking into consideration clinical and experimental findings that show gender differences in the mechanisms involved in pain including CINP and in response to analgesics, it is imperative that future studies on CINP utilise more female models.

## 1. Introduction

Chemotherapy-induced peripheral neuropathy (CIPN) is a dose-limiting side effect of some anticancer drugs, such as bortezomib, cisplatin, oxaliplatin, paclitaxel, thalidomide, and vincristine. The incidence of CIPN in patients varies from as low as 12.1% to as high as 96.2% depending on the chemotherapeutic agent and possibly the type of cancer in which the drug is used. Some chemotherapeutic drugs are associated with high incidence of CIPN; for example, in patients treated with oxaliplatin, it is around 72.3% (95% CI 59.7–86.8) and with paclitaxel it is around 70.8% (95% CI 43.5–98.1) [1]. Some patients who experience CIPN also experience neuropathic pain; for example, amongst patients who experienced CIPN during paclitaxel treatment, 27% of these patients had neuropathic pain [2], and neuropathic pain occurred in the majority of those treated with bortezomib [3]. Some of the symptoms of the painful CIPN, also referred to as chemotherapy-induced neuropathic pain (CINP), include hyperalgesia, allodynia, and spontaneous sensations such as burning, shooting, numbness, spasm, and prickling, and these can significantly reduce the patient's quality of life [4–6]. Regrettably, there is a scarcity of drugs to either prevent or manage CINP. Currently, only duloxetine has a recommendation for the management of CINP, whilst other drugs used for other neuropathic pain conditions may be given because of the limited CINP treatment options [7]. Thus, studies to understand the pathophysiology and to develop novel treatment options for CINP treatment are essential.

Cannabis or cannabinoids have been reported to improve pain measures in patients with neuropathic or cancer pain [8–11]. A recent study recommends studying the effects of a cannabinoid agent, nabiximols, against CINP in a full randomised, placebo-controlled trial [12].

Phytocannabinoids, synthetic cannabinoids, and endogenous cannabinoids (endocannabinoids), such as *N*-arachidonoylethanolamine (AEA, anandamide) and 2-arachidonoylglycerol (2-AG), produce their effects via activation of cannabinoid receptors, CB1 and CB2 receptors [13]. Endocannabinoids are lipid-based neurotransmitters produced on demand [14–17] from cell membrane phospholipid precursors [18, 19] through various pathways [20]. Endocannabinoid homeostasis is maintained by transporters that transport AEA and 2-AG from the synaptic space back into the cells or by enzymes that degrade the endocannabinoids. Fatty acid amide hydrolase (FAAH) hydrolyses AEA to arachidonic acid and ethanolamine [21]. Monoacylglycerol lipase (MGL) hydrolyses 2-AG to arachidonic acid and glycerol [22, 23].

In this minireview, changes that occur in the endocannabinoid system during CINP and the possibility of targeting the endocannabinoid enzymes and cannabinoid receptors to manage CINP will be discussed. This minireview deals only with results obtained from animal studies.

## 2. Methods and Selection of Articles to Include in the Analysis of Endocannabinoid Expression and Compounds Utilising the Endocannabinoid System to Prevent or Treat CINP in Animal Models

The U.S. National Library of Medicine, Washington, DC (MEDLINE-PubMed), was used to search for appropriate papers for this study, without limitations of date of publication, using different combinations of the words or phrases: chemotherapy-induced neuropathy, chemotherapy-induced neuropathic pain, cannabinoid, endocannabinoid, pain, expression, cisplatin, paclitaxel, and vincristine. The search strategy was designed to include any published paper that examined the expression of endocannabinoids in animal models of CINP and/or evaluated the use of cannabinoids or drugs that prevent or treat established hyperalgesia, hypersensitivity, or allodynia in animal models of CINP in a cannabinoid receptor-dependent manner. Articles that were found in more than 1 search combination were counted only once. Studies that did not use animals, did not evaluate CINP, or evaluated noncannabinoid compounds without using cannabinoid receptor antagonists, reviews, commentaries, and *in vitro* studies were excluded.

From the electronic search, a total of 48 articles were identified, and of these, 20 articles were excluded because they were reviews, human studies, *in vitro* studies, did not evaluate CINP, and evaluated noncannabinoid compounds without using cannabinoid receptor antagonists. Twenty-eight articles fulfilled the inclusion and exclusion criteria described above and are discussed in the sections below.

## 3. Expression of Endocannabinoids during Chemotherapy-Induced Neuropathic Pain

Four studies were found that measured the expression of the endocannabinoids 2-AG and AEA in rodent models of CINP [24–27]. All these studies were done in male rodents of cisplatin CINP (Table 1). Treatment of mice with cisplatin reduced the expression of AEA but not 2-AG in the paw skin [25, 27]. Guindon et al. measured the expression of endocannabinoids in the spinal cord and paw skin in rats with cisplatin-induced mechanical and cold allodynia [24]. Rats treated with cisplatin had increased levels of 2-AG and AEA in the lumbar spinal cord, whilst 2-AG, but not AEA, was decreased in the dorsal hind paw skin [24]. In another study, Khasabova et al. showed that treatment of mice with cisplatin resulted in decreased levels of AEA and 2-AG in the paw skin and dorsal root ganglia (DRGs), increased levels of AEA and 2-AG in the spinal cord, but no change in the brain [26]. These studies suggest there is a specific deficiency of endocannabinoids in the periphery during CINP. Thus, increasing the levels of these endocannabinoids in the periphery by inhibiting either their degradation or transport from the synaptic space back into the cells is a potential therapeutic target for managing CINP.

## 4. Effect of Inhibitors of Endocannabinoid Metabolism on Chemotherapy-Induced Neuropathic Pain

Taking into consideration that FAAH and MGL degrade the endocannabinoids, their inhibitors have been evaluated in animal models of CINP. All of the five studies carried out utilised male rodents of CINP (Table 2). An FAAH inhibitor ST4070 suppressed established mechanical allodynia in rats with vincristine-induced mechanical allodynia [28]. Another FAAH inhibitor URB597 delayed the onset and decreased the magnitude of mechanical and heat hyperalgesia and also suppressed established mechanical and thermal hyperalgesia in mice with cisplatin-induced hyperalgesia in a CB1, but not CB2, receptor-dependent manner [25]. Two FAAH inhibitors URB597 and URB937 and an MGL inhibitor JZL184 suppressed established cisplatin-induced mechanical and cold allodynia in rats. The antiallodynic effects of the FAAH inhibitors and MGL inhibitor were blocked by CB1, but not CB2, receptor antagonists [24]. In mice, peripheral administration of low doses of JZL184 was found to prevent the development of cisplatin-induced mechanical hyperalgesia in a CB1 receptor, but not CB2 receptor, dependent manner [26]. URB597 and JZL184 were also reported to suppress paclitaxel-induced mechanical and cold allodynia in mice [29]. These studies show that FAAH and MGL inhibitors can both prevent the development and attenuate established CINP symptoms mainly in a CB1 receptor-dependent manner.

## 5. Effect of Cannabinoid Agonists on Chemotherapy-Induced Neuropathic Pain

The majority of the studies (21 studies) on cannabinoids and CINP in animal models evaluated the effects of agonists via CB1 and CB2 receptors (Table 3). The use of nonselective CB1/CB2 agonists was found in 11 studies, CB1 receptor-selective agonists in 2 studies, and CB2 receptor-selective agonists in 11 studies. Some studies evaluated drugs in more than one of the categories of selectivity for CB receptors. All the studies that stated the gender (19 out of 21) conducted on CB receptor agonists and CINP utilised male rodents (Table 3). Two studies did not state the gender of the mice used (Table 3).

Nonselective CB1/CB2 receptor agonists such as the endocannabinoids 2-AG and AEA, CP55,940, Δ^9^-tetrahydrocannabinol (THC), and WIN 55,212-2 were reported to prevent the development and/or suppress established symptoms of CINP in mice and rats. The endocannabinoids 2-AG and AEA suppressed established cisplatin-induced mechanical hyperalgesia in mice in a CB1, but not CB2, receptor-dependent manner [25, 26]. The phytocannabinoid THC was found to suppress established mechanical and cold allodynia induced by either cisplatin or paclitaxel in mice [30, 31]. However, the role of CB receptors in the activity of THC against CINP was not investigated. THC failed to prevent the development of cisplatin-induced mechanical allodynia [31]. A synthetic nonselective CB receptor agonist CP55,940 suppressed paclitaxel-induced allodynia in mice, via both CB1 and CB2 receptor-dependent mechanisms, but the CB2 receptor-dependent mechanism was only observed at high doses of CP55,940 in CB1 receptor-deficient mice [32]. The nonselective CB receptor agonist used most on CINP in rodents is WIN 55,212-2. WIN 55,212-2 both prevented the development and suppressed established CINP, in a manner which was CB1 and CB2 receptor dependent [33–39]. It has both systemic and local effects; however, its local effects appear to be CB1, but not CB2, receptor dependent [38]. Several studies show that nonselective CB receptor agonists attenuate CINP in a CB1, but not CB2, receptor-dependent manner most likely because CB1 receptors are expressed at higher levels in the nervous system than CB2 receptors, while CB2 receptors are expressed mainly in immune cells and the levels increase in the nervous system during injury. Therefore, when nonselective CB receptor agonists are used, the effects on CB1 receptors are more likely to be predominant.

A CB1 receptor-selective agonist ACEA was found to prevent the development of cisplatin-induced mechanical allodynia in rats, when administered either locally or systemically [38]. However, various problems are associated with CB1 receptor activation such as physical dependence, withdrawal adverse effects, and development of tolerance [29, 30]. A novel CB1 positive allosteric modulator (PAM), GAT211, was found to suppress paclitaxel-induced cold and mechanical allodynia in mice without producing the cardinal signs of CB1 activation, physical dependence or tolerance, which were produced by WIN 55,212-2, an orthosteric cannabinoid agonist [29].

Various CB2 receptor-selective agonists have been reported to prevent the development and/or suppress established symptoms of CINP in mice and rats. The aminoalkylindole cannabinoid AM1241 and the cannabilactones AM1710 and AM1714 were found to prevent the development and suppress established mechanical and cold allodynia induced by cisplatin, paclitaxel, or vincristine in rodents in a CB2, but not CB1, receptor-dependent manner [30, 34, 39, 40, 41]. Other CB2 receptor-selective agonists MDA7, MDA19, JWH133, and LY2828360 were reported to prevent the development and suppress established paclitaxel-induced mechanical and cold allodynia [38, 42–46]. The CB2 receptor-dependent antiallodynic effects were demonstrated by blocking the effects of MDA7 and JWH133 by CB2 receptor antagonists [38, 43], whilst the other studies did not utilise antagonists. LY2828360 suppressed paclitaxel-induced allodynia without producing tolerance [46]. *β*-Caryophyllene (BCP), a CB2 receptor-selective agonist found in many essential oils including clove oil and *Cannabis sativa* essential oil [48], prevented the development and attenuated established paclitaxel-induced mechanical allodynia in mice in a CB2, but not CB1, receptor-dependent manner [47]. Some of the advantages of CB2 receptor-selective agonists such as AM1710, over nonselective CB1/CB2 receptor agonists, such as WIN 55,212-2 and THC, described were the favourable therapeutic ratio because of sustained efficacy and absence of tolerance, physical withdrawal, or CB1-mediated side effects [30]. Therefore, selectively targeting CB2 receptors can produce antiallodynic effects whilst bypassing the unwanted central effects associated with CB1 receptor activation [30].

## 6. Effect of the Nonpsychoactive Phytocannabinoid Cannabidiol on Chemotherapy-Induced Neuropathic Pain

The effects of the nonpsychoactive phytocannabinoid cannabidiol in mice models of CINP were evaluated in 3 studies. Different from the trend noted above, the majority of these studies (two out of three) utilised female mice (Table 4).

Cannabidiol was found to prevent the development of paclitaxel-induced cold and mechanical allodynia [49, 50]. However, in another study, cannabidiol attenuated established cisplatin-induced mechanical allodynia but could not prevent its development [31]. Cannabidiol displays low affinity for CB1 and CB2 receptors but antagonises cannabinoid CB1/CB2 receptor agonists [51]. The antiallodynic effects of cannabidiol were not blocked by either CB1 or CB2 receptor antagonists but were blocked by a 5-HT1A receptor antagonist [50].

## 7. Effect of Other Drugs That Affect the Endocannabinoid System on Chemotherapy-Induced Neuropathic Pain

Other drugs that produce biologic activity via other mechanisms independent of the cannabinoid system have been reported to have antiallodynic and antihyperalgesic activities in animal models of CINP in a cannabinoid receptor-dependent manner [52, 53]. Fifty percent of the studies (one out of two) used female animals (Table 5).

Parvathy and Masocha reported that coadministration of indomethacin plus minocycline attenuates paclitaxel-induced thermal hyperalgesia in mice, whereas the individual drugs did not [53]. The effects of the combination were dependent on CB1 receptors; however, the role of CB2 receptors was not investigated [53]. Indomethacin reduces pain principally through inhibition of cyclooxygenase enzymes, whereas minocycline has anti-inflammatory activities and inhibits lipoxygenases and microglia activation. In addition, these two drugs have been reported to individually modulate the levels of endocannabinoids [54, 55]. Further research is needed to study whether the synergistic effect of coadminstration of indomethacin plus minocycline is via increased levels of endocannabinoids. Coadministration of indomethacin and minocycline previously been reported has more antinociceptive effects in a mouse model of arthritis than when either drug alone was administered [56]. Moreover, indomethacin and minocycline are already clinically used for managing pain and inflammation in humans with conditions such as rheumatoid arthritis [57, 58]. Thus, if confirmed useful in other models of CINP, it would be easier to translate the findings to humans than with synthetic cannabinoid agonists or modulators not yet tried in the clinics.

The role of the cannabinoid receptors in the antiallodynic effects of desipramine was evaluated in mice with paclitaxel-induced mechanical and cold allodynia [52]. Desipramine is a tricyclic antidepressant used clinically in the management of neuropathic pain including CINP [59]. Cannabinoid CB1 and CB2 receptor antagonists partially attenuated the ability of desipramine to prevent the development of paclitaxel-induced mechanical and cold allodynia in these mice [52]. This study adds an understanding of other possible mechanisms of action of desipramine in the management of CINP.

## 8. Concluding Remarks

The endocannabinoid system is dysregulated in animal models of CINP, most notably the reduced levels of AEA and 2-AG in the paw skin, which suggest their deficiency in the periphery but not in the CNS. These findings indicate that inhibitors of FAAH and MGL could result in an elevation of the endocannabinoids and alleviation of CINP. Indeed, FAAH and MGL inhibitors have been found to prevent or attenuate symptoms of CINP in animal models. Closely related to the changes in the expression of endocannabinoids in the periphery, a peripherally acting FAAH inhibitor URB937 alleviated established CINP in rats [24]. Further research on peripherally acting FAAH and MGL inhibitors in the management of CINP is warranted, since they would lack the CNS side effects of centrally acting drugs.

Various agonists of CB1 and CB2 receptors prevent or attenuate symptoms of CINP in animal models; however, various problems are associated with CB1 receptor activation including physical dependence, development of tolerance, and other cannabimimetic adverse effects such as hypothermia and catalepsy. Options to circumvent these problems that have been explored include the use of a CB1 receptor PAM or CB2 receptor-selective agonists. Both the CB1 receptor PAM and CB2 receptor-selective agonists attenuate CINP in animals with less adverse effects than nonselective CB1/CB2 receptor agonists, FAAH and MGL inhibitors. Thus, CB1 receptor PAMs or CB2 receptor-selective agonists warrant further research as they might have a better safety profile than the classical cannabinoids.

Cannabidiol, which is a major constituent of cannabis, attenuates allodynia in animal models of CINP, in part via 5-HT1A receptors but independent of the CB receptors.

Desipramine which is a TCA used clinically in the management of CINP seems to be working partially via CB1 and CB2 receptors. This information aids in the understanding of how some of the drugs currently used to manage CINP work. The study on the coadministration of minocycline and indomethacin shows that the combination attenuates CINP in animals in a CB1 receptor-dependent manner. This study opens an avenue for modulating the endocannabinoid system for managing CINP with drugs that are already being used in the clinics for other conditions.

The studies analysed suggest that targeting the endocannabinoid system for prevention and treatment of CINP is a plausible therapeutic option. Further research is needed to ascertain whether this approach has advantages compared to already existing treatment options for CINP and whether this can be translated into clinical applications.

One striking observation is that the majority of the studies on animal models of CINP analysed in this review utilised male rodents (almost 90%). Current clinical and experimental findings show that there are sex/gender differences in the manifestation and mechanisms involved in pain including CINP [60–63]. Moreover, sex/gender differences in both the expression of endocannabinoids and antinociceptive activity of cannabinoids have been reported [64, 65]. A consensus report published by members of the Sex, Gender and Pain Special Interest Group of the International Association for the Study of Pain recommended that “all pain researchers consider testing their hypotheses in both sexes, or if restricted by practical considerations, only in females” [66]. This is important because of the higher prevalence of some types of pain in females compared to males, the gender differences in response to analgesics both in experimental animals and humans, and the higher number of studies done in male than in female models of pain [66]. Taking this into consideration, it is imperative that future studies on CINP utilise more female models.

## Figures and Tables

**Table 1:** Expression of endocannabinoids in animal models of CINP.

CINP model			Expression of endocannabinoids		Reference
Chemotherapy drug	Animals	CINP symptom	Tissue	Change in endocannabinoid levels relative to control	
Cisplatin	Male C3H/HeN mice	Mechanical and heat hyperalgesia	Plantar paw skin	Decrease in AEA but no change in 2-AG	[25]
Cisplatin	Male SD rats	Mechanical and cold allodynia	Lumbar spinal cord	Both 2-AG and AEA increased	[24]
			Dorsal hind paw skin	2-AG decreased but not AEA	
Cisplatin	Male C3H/HeN mice	Mechanical hyperalgesia	Plantar paw skin	Both 2-AG and AEA decreased	[26]
			DRGs	Both 2-AG and AEA decreased	
			Lumbar spinal cord	Both 2-AG and AEA increased	
			Midbrain	No effect on both 2-AG and AEA	
Cisplatin	Male C3H/HeJ mice	Mechanical allodynia	Plantar paw skin	Decrease in AEA but no change in 2-AG	[27]

2-AG, 2-arachidonoylglycerol; AEA, *N*-arachidonoylethanolamine (anandamide); SD, Sprague-Dawley.

**Table 2:** Effects of inhibitors of enzymes that degrade endocannabinoids in animal models of CINP.

CINP model		Inhibitor of enzymes that degrade endocannabinoids	Effects	Effects of CB receptor antagonists on the activity of the compound	Reference
Chemotherapy drug	Animal				
Vincristine	Male SD rats	FAAH inhibitor ST4070	Suppressed established mechanical allodynia	No antagonists were used against vincristine	[28]
Cisplatin	Male C3H/HeN mice	FAAH inhibitor URB597	Delayed the onset and decreased the magnitude of mechanical and heat hyperalgesia	CB1 antagonist AM281 antagonised	[25]
			Suppressed established mechanical and thermal hyperalgesia	CB2 antagonist AM630 had no effect	
Cisplatin	Male SD rats	FAAH inhibitors URB597 and URB937	Suppressed established mechanical and cold allodynia	CB1 antagonist AM251 antagonised	[24]
		MGL inhibitor JZL184		CB2 antagonist AM630 had no effect	
Cisplatin	Male C3H/HeN mice	MGL inhibitor JZL184	Prevented the development of mechanical hyperalgesia	CB1 receptor antagonist AM281 antagonised	[26]
			Suppressed established mechanical allodynia	CB2 receptor antagonist AM630 had no effect	
Paclitaxel	Male CD1 and C57BL/6J mice	URB597 and JZL184	Suppressed mechanical and cold allodynia	Antagonists were not tested	[29]

FAAH, fatty acid amide hydrolase; MGL, monoacylglycerol lipase; SD, Sprague-Dawley.

**Table 3:** Effects of CB receptor agonists in animal models of CINP.

CINP model		CB receptor agonist	Effects	Effects of CB receptor antagonists on the activity of the compound	Reference
Chemotherapy drug	Animal				
*Nonselective CB1/CB2 receptor agonists*					
Cisplatin	Male C3H/HeN mice	2-AG	Suppressed established mechanical hyperalgesia	CB1 receptor antagonist AM281 antagonised	[26]
				CB2 receptor antagonist AM630 had no effect	
Cisplatin	Male C3H/HeN mice	AEA	Suppressed established mechanical hyperalgesia	CB1 antagonist AM281 antagonised	[25]
				CB2 antagonist AM630 had no effects	
Paclitaxel	CD1 and C57BL/6J mice	THC	Suppressed established mechanical and cold allodynia	No antagonists were used	[30]
Cisplatin	Male C57BL/6 mice	THC	Suppressed established mechanical allodynia	No antagonists were used	[31]
			Did not prevent the development of mechanical allodynia		
Paclitaxel	CD1 and C57BL/6J mice	CP55,940	Suppressed established mechanical and cold allodynia	CB2 antagonist AM630 antagonised	[32]
Paclitaxel	Male Wistar rats	WIN	Suppressed established thermal hyperalgesia and mechanical allodynia	CB1 antagonist SR141716A antagonised	[33]
Vincristine	Male SD rats	WIN	Suppressed established mechanical allodynia	Both CB1 antagonist SR141716 and CB2 antagonist SR144528 antagonised	[34]
Cisplatin	Male Wistar rats	WIN	Prevented the development of mechanical allodynia	No antagonists were used	[35]
Paclitaxel	Male Wistar rats	WIN	Prevented the development of thermal hyperalgesia and mechanical allodynia	No antagonists were used	[36]
Cisplatin	Male Wistar rats	WIN	Prevented the development of mechanical allodynia	No antagonists were used	[37]
Cisplatin	Male Wistar rats	WIN	Suppressed established mechanical allodynia	Both CB1 antagonist AM251 and CB2 antagonist SR144528 antagonised	[38]
Paclitaxel	Male SD rats	WIN	Prevented the development of mechanical and cold allodynia	CB1 receptor antagonist AM251 antagonised mechanical allodynia but not cold allodynia	[39]
				CB2 receptor antagonist AM630 did not reliably antagonise both mechanical and cold allodynia	
*CB1 receptor-selective agonists*					
Cisplatin	Male Wistar rats	ACEA	Suppressed established mechanical allodynia	CB1 antagonist AM251 antagonised	[38]
Paclitaxel	Male CD1 and C57BL/6J mice	GAT211 (CB1 PAM)	Suppressed mechanical and cold allodynia	CB1 receptor antagonist AM251 antagonised	[29]
				CB2 receptor antagonist AM630 had no effect	
*CB2 receptor-selective agonists*					
Vincristine	Male SD rats	AM1241	Suppressed established mechanical allodynia	CB1 antagonist SR141716 had no effect	[34]
				CB2 antagonist SR144528 antagonised	
Paclitaxel	Male SD rats	AM1241 and AM1714	Suppressed established mechanical allodynia	CB1 antagonist SR141716 had no effect	[40]
				CB2 antagonist SR144528 antagonised	
Cisplatin and paclitaxel	Male SD rats	AM1710	Suppressed established mechanical and cold allodynia	CB1 antagonist AM251 had no effect	[41]
				CB2 antagonist AM630 antagonised	
Paclitaxel	Male SD rats	AM1710	Prevented the development of mechanical and cold allodynia	CB1 receptor antagonist AM251 had no effect	[39]
				CB2 receptor antagonist AM630 antagonised	
Paclitaxel	CD1 and C57BL/6J mice	AM1710	Suppressed established mechanical and cold allodynia	CB2 antagonist AM630 antagonised	[30]
Paclitaxel	Male SD rats	MDA7	Suppressed established mechanical allodynia	No antagonists were used	[42]
Paclitaxel	Male SD rats and mice (strain not specified)	MDA7	Suppressed established mechanical allodynia	CB2 antagonist AM630 antagonised	[43]
Paclitaxel	Male SD rats	MDA7	Prevented the development of mechanical allodynia	No antagonists were used in paclitaxel-induced mechanical allodynia	[44]
Paclitaxel	Male SD rats and mice (strain not specified)	MDA19	Suppressed established mechanical allodynia	No antagonists were used	[45]
Paclitaxel	Male C57BL/6J mice	LY2828360	Suppressed established mechanical and cold allodynia	No antagonists were used	[46]
Cisplatin	Male Wistar rats	JWH133	Suppressed established mechanical allodynia	CB2 antagonist SR144528 antagonised	[38]
Paclitaxel	Male Swiss mice	BCP	Prevented the development of mechanical allodynia	CB1 receptor antagonist AM251 had no effect	[47]
			Suppressed established mechanical allodynia	CB2 receptor antagonist AM630 antagonised	

AEA, *N*-arachidonoylethanolamine (anandamide); BCP, *β*-caryophyllene; PAM, positive allosteric modulator; SD, Sprague-Dawley; THC, Δ^9^-tetrahydrocannabinol; WIN, WIN 55,212-2.

**Table 4:** Effects of cannabidiol in animal models of CINP.

CINP model		Effects	Effects of CB receptor antagonists on the activity of the compound	Reference
Chemotherapy drug	Animal			
Paclitaxel	Female C57Bl6 mice	Prevented the development of cold and mechanical allodynia	No antagonists were used	[49]
Paclitaxel	Female C57Bl6 mice	Prevented the development of mechanical allodynia	CB1 receptor antagonist SR141716 and	[50]
			CB2 receptor antagonist SR144528 had no effects	
			5-HT1A receptor antagonist WAY 100635 antagonised	
Cisplatin	Male C57BL/6 mice	Suppressed established mechanical allodynia	No antagonists were used	[31]
		Did not prevent the development of mechanical allodynia		

**Table 5:** Effects of other agents, in a CB receptor-dependent manner, in animal models of CINP.

CINP model		Molecule	Effects	Effects of CB receptor antagonists on the activity of the compound	Reference
Chemotherapy drug	Animal				
Paclitaxel	Female and male BALB/c mice	Indomethacin (NSAID) plus minocycline (semisynthetic tetracycline antibiotic)	Reduced established thermal hyperalgesia	CB1 antagonist AM251 antagonised	[53]
Paclitaxel	Male Sprague-Dawley rats	Desipramine (tricyclic antidepressant)	Prevented the development of mechanical and cold allodynia	CB1 antagonist AM251 and CB2 antagonist AM630 partially antagonised	[52]

NSAID, nonsteroidal anti-inflammatory drug.
